# Chromophore of an Enhanced Green Fluorescent Protein Can Play a Photoprotective Role Due to Photobleaching

**DOI:** 10.3390/ijms22168565

**Published:** 2021-08-09

**Authors:** Joanna Krasowska, Katarzyna Pierzchała, Agnieszka Bzowska, László Forró, Andrzej Sienkiewicz, Beata Wielgus-Kutrowska

**Affiliations:** 1 Division of Biophysics, Institute of Experimental Physics, Faculty of Physics, University of Warsaw, Pasteura 5, 02-093 Warsaw, Poland; Joanna.Krasowska@fuw.edu.pl (J.K.); Agnieszka.Bzowska@fuw.edu.pl (A.B.); 2 Laboratory for Functional and Metabolic Imaging (LIFMET), Institute of Physics (IPHYS), School of Basic Sciences (SB), Ecole Polytechnique Fédérale de Lausanne (EPFL), CH-1015 Lausanne, Switzerland; katarzyna.pierzchala@epfl.ch; 3 Laboratory of Physics of Complex Matter (LPMC), Institute of Physics (IPHYS), School of Basic Sciences (SB), Ecole Polytechnique Fédérale de Lausanne (EPFL), CH-1015 Lausanne, Switzerland; laszlo.forro@epfl.ch; 4 Laboratory for Quantum Magnetism (LQM), Institute of Physics (IPHYS), School of Basic Sciences (SB), École Polytechnique Fédérale de Lausanne (EPFL), Station 3, CH-1015 Lausanne, Switzerland; 5 ADSresonances, Route de Genève 60B, CH-1028 Préverenges, Switzerland

**Keywords:** EGFP, photoprotection, superoxide radicals, singlet oxygen, scavenger, electron spin resonance, spin trapping, reactive oxygen species

## Abstract

Under stress conditions, elevated levels of cellular reactive oxygen species (ROS) may impair crucial cellular structures. To counteract the resulting oxidative damage, living cells are equipped with several defense mechanisms, including photoprotective functions of specific proteins. Here, we discuss the plausible ROS scavenging mechanisms by the enhanced green fluorescent protein, EGFP. To check if this protein could fulfill a photoprotective function, we employed electron spin resonance (ESR) in combination with spin-trapping. Two organic photosensitizers, rose bengal and methylene blue, as well as an inorganic photocatalyst, nano-TiO_2_, were used to photogenerate ROS. Spin-traps, TMP-OH and DMPO, and a nitroxide radical, TEMPOL, served as molecular targets for ROS. Our results show that EGFP quenches various forms of ROS, including superoxide radicals and singlet oxygen. Compared to the three proteins PNP, papain, and BSA, EGFP revealed high ROS quenching ability, which suggests its photoprotective role in living systems. Damage to the EGFP chromophore was also observed under strong photo-oxidative conditions. This study contributes to the discussion on the protective function of fluorescent proteins homologous to the green fluorescent protein (GFP). It also draws attention to the possible interactions of GFP-like proteins with ROS in systems where such proteins are used as biological markers.

## 1. Introduction

The problem of photodamage caused by reactive oxygen species (ROS) and the importance of photoprotection of biomolecular systems against such damage have been the subjects of considerable debate for many years. ROS are formed continuously within living cells during various metabolic processes. However, an uncontrolled rise in ROS levels has harmful effects on cellular homeostasis and can lead to oxidative stress, which, in turn, results in serious irreversible damage to biomolecules in living organisms [1,2,3,4,5].

Living cells are equipped with multiple defense systems against oxidative damage, including enzymatic and non-enzymatic antioxidants, such as superoxide dismutase (SOD) and catalase (CAT), glutathione peroxidase (GPX) and reductase (GSH), melatonin, coenzyme Q, as well as metal-chelating proteins, which can also neutralize excessive ROS [6].

In this context, it has been put forward that naturally occurring fluorescent proteins, such as those found in marine organisms of reef-forming corals and jellyfish, can play an important biological role. In fact, the photoprotective function of the green fluorescent protein (GFP) from *Acropora yongei* and the red fluorescent protein (amilFP597) from *Acropora millepora*, has been already discussed based on in vivo studies [7,8]. In particular, it has been observed that the concentration of these pigments in the host organism reversibly changed as a function of light intensity. It has been then concluded that the high-level expression of fluorescent proteins is correlated with reduced photodamage, which supports the hypothesis claiming the photoprotective function of these molecules. Moreover, Palmer et al. observed a positive correlation between H_2_O_2_ scavenging rate and concentrations of fluorescent proteins in corals [9], while Leutenegger et al. found that properties of GFP-like proteins made them well suited to fulfill photoprotection of biological organisms from damage caused by excessive light [10].

**Figure 1:**
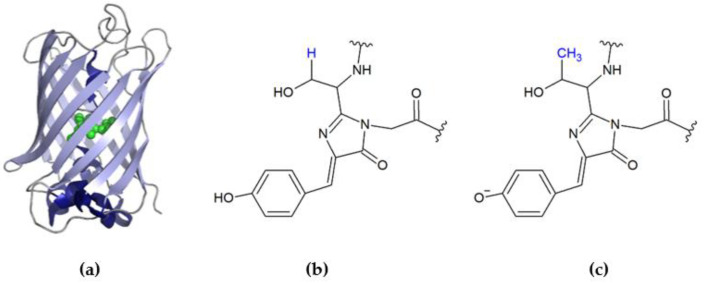
(**a**) The structure of EGFP (PDB ID 2Y0G [13]) with the highlighted location of the chromophore (in green). (**b**) The molecular structure of the wild type GFP chromophore (PDB ID 1W7S [12]) with Ser at 65 position. (**c**) The molecular structure of the EGFP chromophore with Thr at 65 position. The three-dimensional structure of EGFP and the molecular chemical structures of the chromophores were rendered using the public access software, Pymol v0.99 and ACD/ChemSketch, respectively.

The first reported fluorescent protein (FP) was the wild-type green fluorescent protein (GFP), which was isolated by Osamu Shimomura from the Pacific jellyfish, *Aequorea victoria* [11]. The resolved molecular structure of recombinant GFP [12] reveals that the protein is in the shape of a cylinder (β-barrel), which is composed of 11 β-strands arranged mostly in the antiparallel fashion. The hydrogen bonds between adjacent β-strands allow for the formation of an enclosed structure with an α-helical segment buried inside (Figure 1a). Three residues in this segment (Ser65, Tyr66, and Gly67 in GFP [12] or Thr65, Tyr66, and Gly67 in EGFP [13]) participate in an autocatalyzed multistage reaction (cyclization, oxidation, and dehydration [14,15]), which, in the presence of molecular oxygen, generates the p-hydroxybenzylidene-imidazolidone chromophore (Figure 1b,c).

The GFP chromophore, responsible for the fluorescence in the green region of visible light, can exist either in its neutral (protonated) or anionic (deprotonated) form. The neutral form absorbs in the ultraviolet at ~395 nm, whereas the anionic form absorbs at ~475 nm. The serine-65 into threonine mutation (S65T) rearranges the hydrogen bond pattern of glutamic acid-222, suppressing its negative charge, allowing the chromophore to exist mainly in the anionic form [16].

Because of slow folding, low solubility, and slow chromophore maturation of the wild type GFP [17], a number of modifications have been proposed to improve the relevant properties of proteins belonging to the GFP-family. In particular, by mutating the phenylalanine-64 to leucine (F64L) and the chromophore residue serine-65 to threonine (S65T), an enhanced GFP (EGFP) was obtained, with the excitation maximum shifted from ultraviolet to blue and with better folding efficiency in *E. coli* [18]. Excitation in the blue region of the visible spectrum is considered advantageous because it matches up with the wavelengths of lasers, which are often employed in modern cell sorting machines [19].

EGFP with absorption and emission maxima, at 489 nm and 509 nm, respectively, exhibits significantly increased fluorescence efficiency. In particular, due to the preferred anionic form and improved brightness, the latter defined as a product of extinction coefficient and quantum yield [20], being 22,800 M^−1^cm^−1^ and 7500 M^−1^cm^−1^ for EGFP and wild-type GFP, respectively (based on [21,22]), EGFP is often used as a fluorescent intracellular marker in bioimaging in vitro and in vivo.

Although the naturally occurring GFP does not participate in biochemical reactions in living cells, therefore being considered nontoxic and commonly used as a neutral biological marker [23], some engineered fluorescent proteins, with modified structures and changed properties, may reveal harmful cytotoxic effects. In this regard, contrary to the herein proposed photoprotective role of fluorescent proteins in marine organisms, a fluorescent protein derived by mutation from GFP and suitably named ‘KillerRed’, has been found phototoxic upon illumination with light of 540–580 nm due to the formation of ROS [24]. It has been demonstrated that the main photogenerated ROS by ‘KillerRed’ are superoxide radicals (O_2_^•−^) and hydrogen peroxide (H_2_O_2_) [25,26]. The mechanism of production of these reactive species runs through the stage of direct electron transfer from the excited state chromophore to molecular oxygen, thus leading to the formation of O_2_^•−^, which, in turn, through further secondary reactions, generate other forms of ROS [27]. The strong phototoxicity of ‘KillerRed’ is likely due to a combination of its structural properties: (i) a long water-filled ordered channel, which facilitates the access of solvent to the reactive groups of the chromophore, and (ii) a precise amino acid configuration able to stabilize the chromophore in its excited state [28]. Additionally, TagRFP [29] and SuperNova [30] can generate ROS upon irradiation and can be together with KillerRed [24] used for inactivation of specific proteins by chromophore-assisted light inactivation (CALI) in vivo [24,29,30].

Summarizing, fluorescent proteins are capable of both generating and quenching ROS. In this context, to better understand the relevant properties of EGFP, we implemented electron spin resonance (ESR) in combination with spin-trapping. In particular, to gather more insight into the potential ROS generation or quenching capabilities of EGFP in the aqueous environment in vitro, we employed the commonly used spin traps, such as TMP-OH and DMPO, as well as a highly water-soluble ‘stable’ nitroxide radical, TEMPOL. These compounds served as molecular targets for ROS, which were photogenerated in the presence of two organic photosensitizers, rose bengal (RB) and methylene blue (MB), as well as of a commercial inorganic photocatalyst, titanium dioxide (nano-TiO_2_, AMT-100) from Tayca corp. Depending on the photogeneration system used, the ability of EGFP to scavenge ROS was characterized under visible (VIS) or near ultraviolet (UVA) light illumination. We have also compared the ROS quenching capability of EGFP with the relevant abilities of other proteins, such as purine nucleoside phosphorylase (PNP), bovine serum albumin (BSA), and papain. While avoiding direct comparison with superoxide dismutase (SOD), a well-known and very powerful superoxide radical scavenger [31], the significant ROS quenching ability was attributed to EGFP, which might suggest the photoprotective role of this protein in vivo.

Briefly, the data presented herein bring an additional input into the discussion on photoprotective versus phototoxic functions of EGFP. Therefore, the results of this work can be important in research on the photoprotective role of fluorescent proteins against free radicals generated by light (visible or UV). Moreover, they also should be taken into account in other areas, such as, e.g., fluorescence techniques implementing fluorescent proteins as light-excited markers.

## 2. Results

### 2.1. EGFP Is Not a ROS Generator

The prerequisite for this work was to check whether EGFP itself was a ROS photosensitizer under a visible light illumination. For this purpose, we performed ESR spin-trapping measurements for an aqueous EGFP solution using two different spin-traps, which can serve as efficient ROS scavengers to produce more stable spin-adducts, thus facilitating the ESR detection of short-lived forms of ROS. The first of them, DMPO, is routinely used for scavenging hydroxyl (OH^•^) and superoxide radicals (O_2_^•−^), while the second, TMP-OH, is used as a scavenger of singlet oxygen ^1^O_2_ (^1^Δ_g_). The molecular structures of these two diamagnetic spin-traps (TMP-OH and DMPO) and ROS scavenger TEMPOL are presented in Appendix A (Scheme A2), together with their primary reactions with ROS.

**Figure 2:**
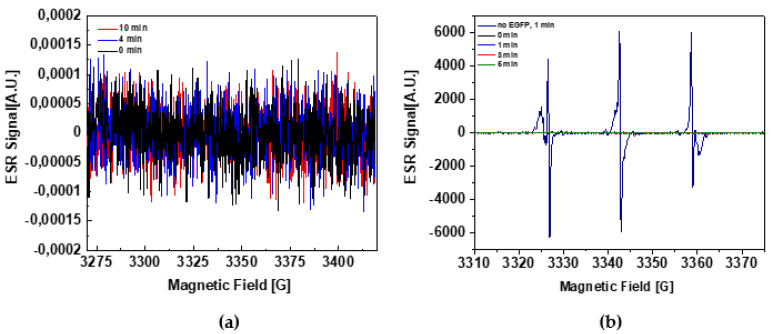
Test measurements performed for checking the potential ability of EGFP to photogenerate ROS under visible light illumination. (**a**) Superimposed are three ESR traces collected for an aqueous solution of EGFP after the consecutive periods of exposure to a visible light of 0 (black), 4 (red), and 10 (blue) minutes, in the presence of DMPO spin-trap. The fact that ESR signals characteristic of the trapped radicals are not observed suggests the lack of ROS photogeneration in the presence of EGFP. In this experiment, 50 μM EGFP was dissolved in the phosphate buffer (pH 8.0), which also contained 300 mM NaCl and a spin-trap, DMPO (50 mM). (**b**) Typically observable rapid increase of the complex TEMPOL/TEMPONE signal during photogeneration of ROS in D_2_O in the presence of 100 μM rose bengal and 50 mM TMP-OH (navy blue) is compared to three superimposed ESR traces acquired in the presence of 100 μM EGFP, after the consecutive periods of exposure to a visible light of 1 (blue), 3 (red), and 5 (green) minutes. The second experiment was intentionally performed in D_2_O to enhance the impact of the potentially possible photosensitization of ^1^Δ_g_ by EGFP. In both experiments, for visible light illumination a spot halogen source (150 W) was used.

The solution was exposed to visible light (150 W halogen source) for 0, 2, 4, and 10 min and for 0, 1, 3, and 5 min in experiments with DMPO and TMP-OH, respectively. As can be seen in Figure 2, in the presence of EGFP, none of these experiments showed the appearance and a measurable growth of signals typical for ROS scavenging, i.e., DMPO-OH/DMPO-OOH and TEMPOL, for DMPO and TMP-OH, respectively.

Thus, the obtained results suggest that EGFP is not a direct ROS photosensitizer under visible light illumination.

### 2.2. ROS Quenching by EGFP

**Figure 3:**
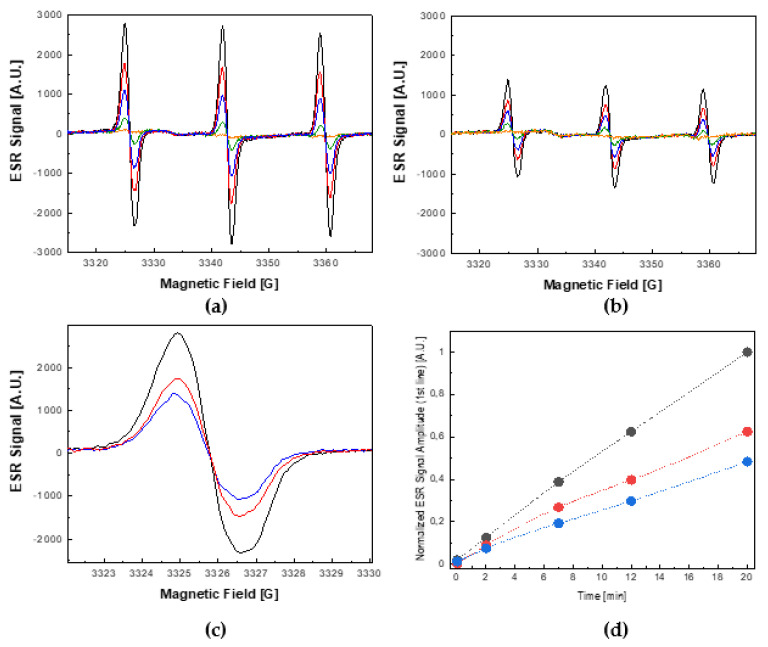
Photosensitized generation of a singlet oxygen (^1^Δ_g_) by 100 μM methylene blue under visible light illumination (150 W halogen) in the absence and presence of EGFP. (**a**) Growth of the ESR signal of TEMPOL as a function of illumination time in the absence of EGFP. (**b**) Growth of the ESR signal of TEMPOL as a function of illumination time in the presence of 20 μM EGFP. The ESR spectra presented in panels (**a**,**b**) were recorded in the dark (orange) and after 2 (green), 7 (blue), 12 (red), and 20 (black) min of visible light illumination. (**c**) Overlaid low-field features of the ESR signal of TEMPOL collected after 20 min of visible light illumination for the photosensitization processes performed: in the absence of EGFP (black trace), and in the presence of 10 μM EGFP (red trace) and 20 μM EGFP (blue trace). (**d**) Time-evolution of the signal amplitude of the low-field feature of the ESR spectra of TEMPOL observed in the absence of EGFP (black dots) and in the presence of 10 μM EGFP (red dots) and 20 μM EGFP (blue dots).

**Scheme A1:**
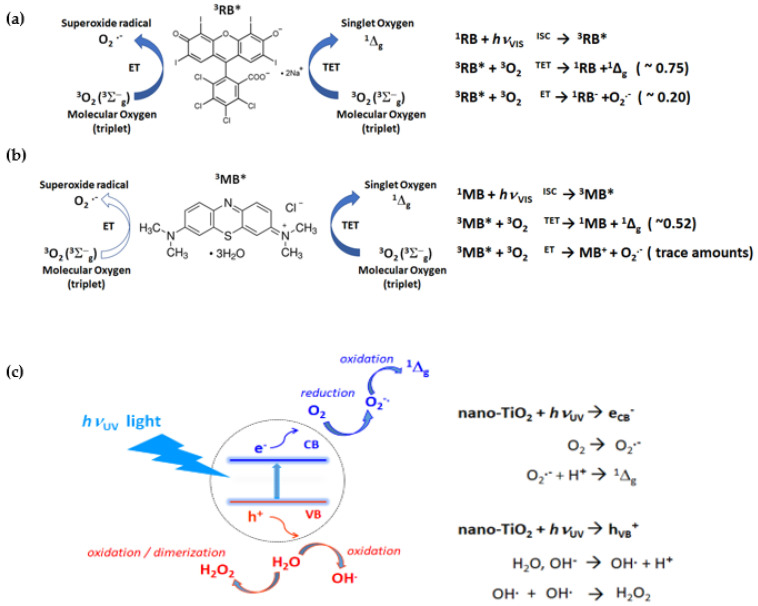
(Left) Structures of organic and inorganic photosensitizers used in this study, i.e., of rose bengal, RB (**a**), methylene blue, MB (**b**) and nano-TiO_2_, (**c**). (Right) Relevant reaction schemes of ROS generation processes. ET, electron transfer; TET, triplet–triplet energy transfer; ISC, inter system crossing; CB, conduction band; h^+^, positively charged holes; VB, valence band.

Next, the ROS quenching capability of EGFP was verified for ROS, which were photogenerated using either one of the organic photosensitizers, such as rose bengal (RB) and methylene blue (MB), or in aqueous suspensions of the nanocrystalline titanium dioxide (nano-TiO_2_, AMT-100). Structures and primary ROS production pathways for organic and inorganic photosensitizers used herein upon excitation with visible or UV light are presented in Appendix A (Scheme A1). In typical experiments, the concentrations of scavengers were about 1000 times higher than the concentrations of ROS generators. The results of the experiment aimed at testing the ability of EGFP to scavenge ROS photogenerated by a visible light excited MB are shown in Figure 3.

Upon excitation, MB generates singlet oxygen (^1^Δ_g_) (see Scheme A1b). Therefore, in this experiment, the ESR-silent scavenger, TMP–OH, was used, which, upon reacting with the photosensitized ^1^Δ_g_, turns into an ESR-active nitroxide radical, TEMPOL. The ESR spectra were recorded in the dark and after the consecutive periods of exposure to visible light of 2, 7, 12, and 20 min, in the absence or presence of EGFP.

In particular, the progressive growth of the ESR signal of TEMPOL as a function of the illumination time in the absence and presence of EGFP is shown in Figure 3a,b, respectively. It can be seen that the resultant ESR signal after 20 min of illumination in the presence of EGFP was ~50% lower (Figure 3b) than the corresponding ESR signal acquired in the absence of EGFP (Figure 3a). Moreover, as shown in Figure 3c,d, the observed damping effect of EGFP increased in a concentration-dependent manner. This result suggests that ^1^Δ_g_, which is the main ROS photosensitized by MB under a visible light illumination, was partially quenched by EGFP.

Moreover, as can be deduced from Figure 3c, which shows the overlaid low-field hyperfine features of the ESR spectra of TEMPOL collected after 20 min of exposure to visible light for three concentrations of this protein (0, 10, and 20 μM), the shape of the ESR spectrum points to the exclusive formation of TEMPOL. In other words, it indicates the exclusive presence of ^1^Δ_g_ in this experiment, thus excluding formation and interference of other ROS, such as O_2_^•−^ and OH^•^, which would modify the ESR signal by an admixture of the ESR signal of TEMPONE.

**Figure 4:**
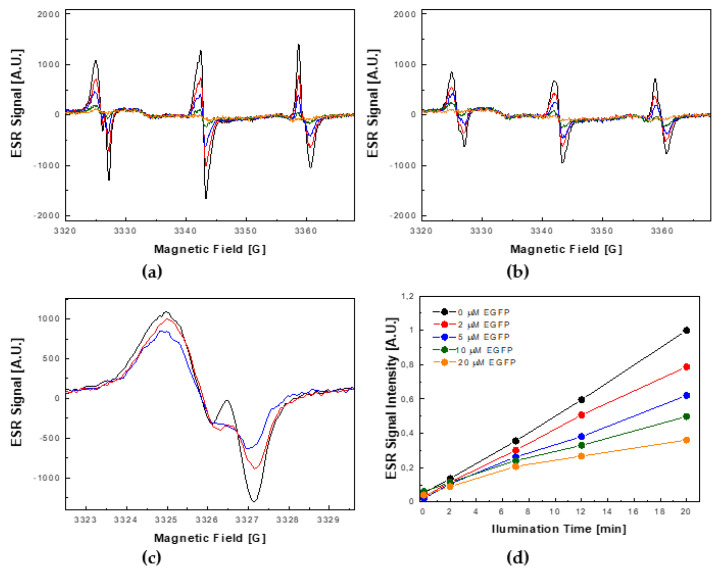
Photosensitized generation of a singlet oxygen (^1^Δ_g_) and superoxide radicals (O_2_^•−^) by 50 μM rose bengal under visible light illumination in the absence and presence of EGFP. (**a**) Growth of the complex ESR signal containing the components of TEMPOL and TEMPONE, as a function of the illumination time in the absence of EGFP. (**b**) Growth of the complex ESR signal containing the components of TEMPOL and TEMPONE as a function of the illumination time in the presence of 5 μM EGFP. The ESR spectra presented in panels (**a**,**b**) were recorded in the dark (orange) and after 2 (green), 7 (blue), 12 (red), and 20 (black) min of visible light illumination. (**c**) Overlaid low-field features of the complex ESR signal acquired after 20 min of visible light illumination for the photosensitization processes performed in the absence of EGFP (black trace) and in the presence of 2 μM EGFP (red trace) and 5 μM EGFP (blue trace). (**d**) Time-evolutions of the signal amplitude of the low-field feature of the ESR spectra of TEMPOL observed in the absence of EGFP (black dots) and in the presence of 2 μM EGFP (red dots), 5 μM EGFP (blue dots), 10 μM EGFP (green dots), and 20 μM EGFP (orange dots).

A similar experiment, aiming at testing the ability of EGFP to simultaneously scavenge singlet oxygen (^1^Δ_g_) and superoxide radicals (O_2_^•−^), was also performed. To that end, RB was used because it is a visible light-sensitive photosensitizer that generates both ^1^Δ_g_ and O_2_^•−^, with the respective efficiencies of ~75% and ~20% (see Scheme A1a). The results of this experiment are shown in Figure 4.

**Scheme A2:**
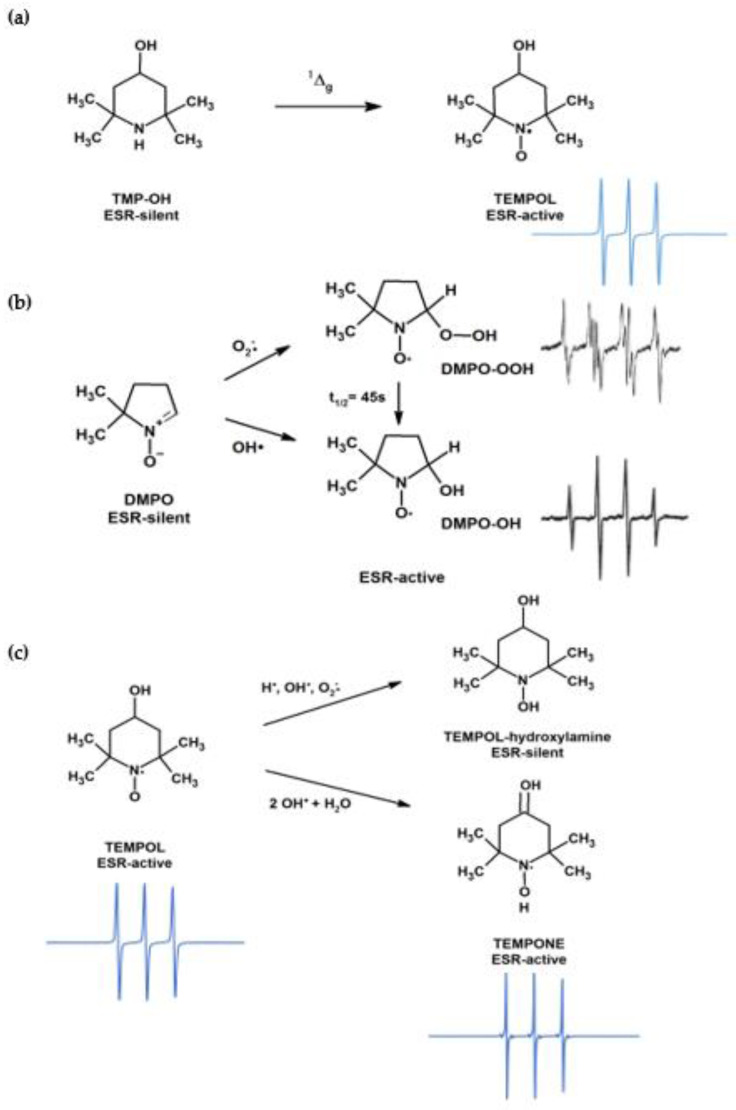
Schematic representation of typical reactions of ROS with the spin-traps, TMP-OH and DMPO. (**a**) Scavenging of excited singlet oxygen (^1^Δ_g_) by TMP-OH results in appearing of the ESR signal of TEMPOL. (**b**) Scavenging of hydroxyl radicals (OH^•^) by DMPO results in formation of the paramagnetic spin-adduct, DMPO-OH. Scavenging of superoxide radicals (O_2_^•−^) by DMPO leads to the short-lived paramagnetic spin-adduct DMPO-OOH, which converts to DMPO-OH. (**c**) Possible reaction pathways of TEMPOL with ROS. The attack of hydroxyl radicals (OH^•^) converts TEMPOL to TEMPONE, whereas reactions with other ROS lead to formation of the ESR-silent products.

The overall behavior of the ESR signal was similar to that observed in the experiment using MB as a photosensitizer. In particular, in the absence of EGFP, due to scavenging of ^1^Δ_g_ by TMP-OH, the ESR signal linearly increased as a function of time after the consecutive periods of exposure to visible light illumination (Figure 4a). Additionally, similarly to the experiment using MB, the increase of this signal was markedly weakened in the presence of EGFP (Figure 4b). However, although the overall time-evolutions of the observed signals in both experiments were similar, the spectral shapes of ESR signals collected for the photosensitization process with RB were distinctly different. Specifically, due to the simultaneous action of both ^1^Δ_g_ and O_2_^•−^, the growth of the TEMPOL signal was also accompanied by the appearance of three components of a weaker and substantially narrower signal, which could be assigned to TEMPONE. In general, in experiments utilizing TMP-OH as a molecular target, the appearance of the ESR signal of TEMPONE indicates involvement of other forms of ROS than just ^1^Δ_g_ [32]. This statement is consistent with the proposed reaction mechanisms of TEMPOL with O_2_^•−^ and OH^•−^ that lead to the formation of TEMPONE (see Appendix A, Scheme A2c).

### 2.3. Photoprotective Role of EGFP in the Presence of Photocatalytically Generated ROS by Nano-TiO_2_

In another system aiming to investigate the photoprotective role of EGFP we implemented an inorganic photocatalyst, namely nano-TiO_2_. It is widely accepted that under exposure to ultraviolet radiation, the nanocrystalline form of titanium dioxide efficiently generates both O_2_^•−^ and OH^•−^ (see Appendix A, Scheme A1c). Therefore, for this experiment, the commercially-available nano-TiO_2_ AMT-100 was chosen as a UVA-light-active inorganic photocatalyst. This material consists of anatase nanoparticles having an average diameter of ~6 nm and is also characterized by a very large specific surface area of ~280.0 m^2^/g.

To detect photocatalytically generated ROS, we implemented a spin-trap, DMPO. The measurements were performed in the absence and presence of 3.6 μM EGFP. In addition, a virtually identical process of photocatalytic ROS generation by nano-TiO_2_ was carried out in the presence and absence of superoxide dismutase (SOD) with the activity of 30 U/mL, a very powerful naturally-occurring antioxidant enzyme [33], and in the presence and absence of bovine serum albumin (BSA), which is also known as a potent antioxidant [34,35].

**Figure 5:**
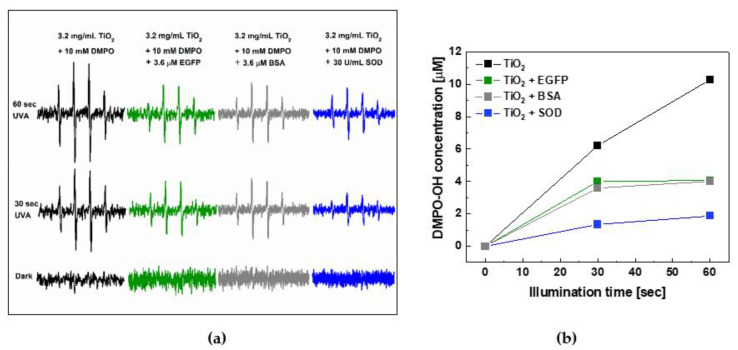
The ROS quenching ability of EGFP towards photocatalytically generated ROS in the presence of 10 mM DMPO and nano-TiO_2_, AMT-100 (3.2 mg/mL) in comparison with SOD and BSA, under UVA illumination (λ = 365 nm, 20 mW/cm^2^). (**a**) Typical ESR spectra of the DMPO-OH spin adduct acquired in the absence of proteins (black), the presence of 3.6 μM EGFP (green) or 3.6 μM BSA (grey), and the presence of 30 U/mL of a superoxide radical scavenging protein, SOD, obtained in the dark and after 30 and 60 s of UVA illumination. (**b**) The time-evolution plots acquired in the absence of protein (black), the presence of EGFP (red), BSA (grey), and SOD (blue).

Typical results of the detection of ROS generated by nano-TiO_2_ in the presence and absence of EGFP, BSA and SOD, without illumination and after 30 and 60 s of exposure to UVA, are shown in Figure 5a. The corresponding time-evolution of the ESR signal intensities is shown in Figure 5b. It can be noticed that in the presence of 3.6 μM EGFP, the intensity of the DMPO-OH signal, confirming the ROS generation, decreased by more than 50% after 60 s of UVA illumination, thus similarly as in the case of BSA with the same concentration.

Overall, the results of this experiment, in which ROS was photocatalytically generated by an inorganic photocatalyst, nano-TiO_2_, point to a significant similarity of the photoprotective behaviors of EGFP and BSA, whereas SOD showed the expected highest quenching performance.

### 2.4. The Comparison of ROS Quenching Abilities of EGFP with PNP and Papain

To check if the ability to quench ROS is a specific feature of EGFP, we compared its ROS scavenging ability with that of two other proteins, purine nucleoside phosphorylase (PNP) and papain. In this context, it is worth adding that while the radical scavenging activity and photoprotective role of papain has been mentioned in the literature [36], data on similar properties of PNP are not known.

Therefore, our experiment was intended to test the ability of the above-mentioned three proteins to intercept two kinds of ROS, which are singlet oxygen (^1^Δ_g_) and superoxide radicals (O_2_^•−^). For this purpose, RB was chosen as a photosensitizer, because it is known that it generates both these ROS species [37].

A control measurement was performed for a solution containing no proteins. The resultant ESR signals were measured for all the systems after 20 min of exposure to visible light. Total concentrations of detected spins were obtained by double integration of recorded ESR signals and by comparing them with the reference signal of 50 μM TEMPOL.

**Figure 6:**
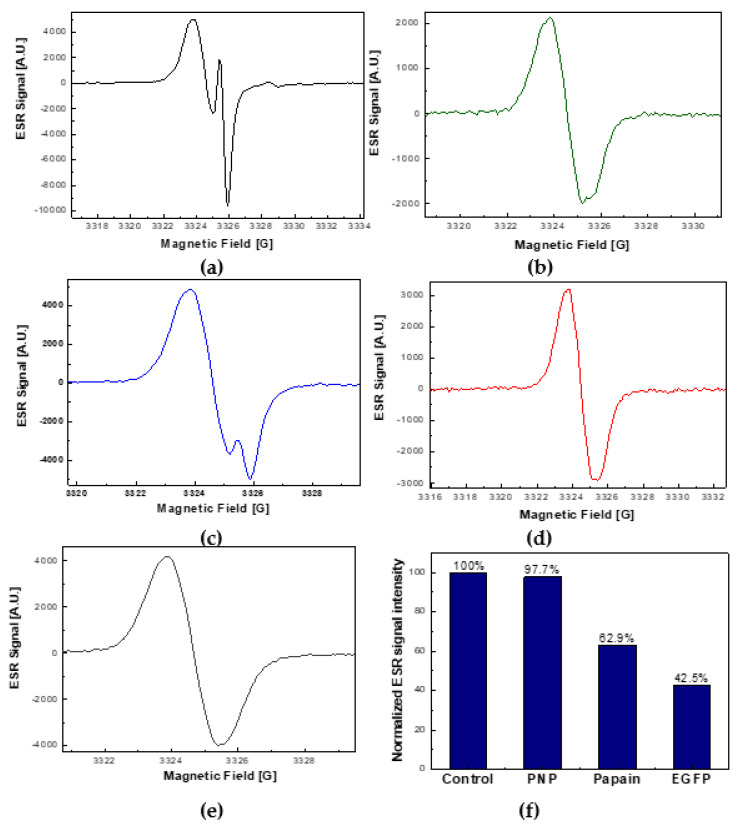
Comparison of the ROS quenching capacity of three proteins, PNP, papain, and EGFP, during the photosensitized generation of singlet oxygen (^1^Δ_g_) and superoxide radicals (O_2_^•−^) by 50 μM rose bengal exposed to visible light. Under the action of photosensitized ROS, the spin-trap TMP-OH (50 mM) is converted to the ESR-active nitroxide radicals, TEMPOL and TEMPONE. (**a**) The low-field hyperfine ESR feature acquired for the control measurement in the absence of proteins. (**b**) The low-field hyperfine ESR feature acquired in the presence of 40 μM EGFP. (**c**) The low-field hyperfine ESR feature acquired in the presence of 40 μM PNP. (**d**) The low-field hyperfine ESR feature acquired in the presence of 32 μM papain. (**e**) The low-field hyperfine ESR feature collected for a reference sample (50 μM TEMPOL). (**f**) Juxtaposition of the normalized ESR signal intensities recorded for the three tested proteins after 20 min of exposure to visible light. All ESR spectra were recorded using the same experimental settings (0.63 mW microwave power, 0.5 G mod., 4 scans per trace).

The comparative results of the ROS scavenging efficiency for the three above-mentioned proteins are shown in Figure 6.

In particular, Figure 6 shows very large spectral differences that can be observed for the ESR signals collected after a 20-min exposure time to visible light of the control sample (Figure 6a) and for the three proteins: EGFP (Figure 6b), PNP (Figure 6c), and papain (Figure 6d). It can be seen that that the low-field features of the corresponding ESR signals differed both in their intensity and spectral shape. Specifically, in the absence of proteins, a typical complex ESR signal consisting of two components, i.e., TEMPOL and TEMPONE, was observed. The stronger component of the signal (TEMPOL) corresponded to the singlet oxygen (^1^Δ_g_) capturing mechanism, whereas the appearance of a weaker component (TEMPONE) indicated the mechanism of capturing superoxide radicals (O_2_^•−^) by TMP-OH (see Scheme A2c). Simulation of the signal depicted in Figure 6a indicated the relative contents of 90% and 10%, for TEMPOL and TEMPONE, respectively (Supplementary Figure S1).

In contrast, for the sample containing 40 μM EGFP, apart from a general decrease in the ESR signal intensity (by ~58%), the characteristic TEMPONE component was practically absent (Figure 6b). A similar signal shape, with similarly reduced intensity (by ~37%), could be observed for the sample containing 32 μM papain (Figure 6d). From the point of view of the line-shape, the ESR signals collected in the presence of these two proteins, EGFP and papain, resembled the TEMPOL signal itself, which is shown for comparison in Figure 6e. In contrast, the admixture of the TEMPONE component, although quite small, was clearly visible in the signal collected for the sample containing 40 μM PNP (Figure 6c).

The comparison of the normalized intensities of ESR signals collected after 20 min of illumination with visible light for the three proteins is shown in Figure 6f. It can be seen that in the presence of EGFP, the intensity of the ESR signal reached the lowest value (42.5% as compared to the control signal). Therefore, considering the above measurements carried out for three proteins, it can be stated that it is EGFP that has the greatest ROS scavenging ability. Additionally, taking into account the practical absence of the TEMPONE component in the resultant ESR signals for EGFP and papain, it can also be stated that these two proteins capture superoxide radicals (O_2_^•−^) more efficiently than singlet oxygen (^1^Δ_g_) (Supplementary Figure S2).

It is worth mentioning that an additional experiment enabled us also to compare the ROS-quenching potential of EGFP, PNP, and papain with the corresponding properties of BSA. A detailed description of this experiment, based on experimental conditions identical to the above-presented, can be found in Supplementary Materials (Section 3). The obtained results suggest that under experimental conditions used herein, the ROS quenching ability runs from the highest to the lowest in the following way: BSA > EGFP > papain > PNP (see Supplementary Figure S3f).

### 2.5. Photobleaching of EGFP Chromophore

To confirm that the EGFP protein chromophore is responsible for the ROS quenching by the process of chromophore photobleaching, we performed additional measurements of ROS-induced decay of the chromophore fluorescence. The experiment was carried out under visible light illumination using MB as photosensitizer. The choice of MB (λ_abs_^max^/λ_em_^max^ = 665 nm/686 nm), instead of RB (λ_abs_^max^/λ_em_^max^ = 546 nm/567 nm) as a ROS photosensitizer, allowed the potential fluorescence resonance energy transfer (FRET) between EGFP (λ_abs_^max^/λ_em_^max^ = 489 nm/509 nm) and RB to be prevented, which may occur due to a partial overlap of the EGFP emission and RB absorption spectra. For the purposes of these measurements, a 10 μM solution of EGFP in phosphate buffer (pH 8.0, 300 mM NaCl), also containing 100 μM MB and 50 mM TMP-OH, was prepared. The control solution contained the same ingredients except MB and TMP-OH.

**Figure 7:**
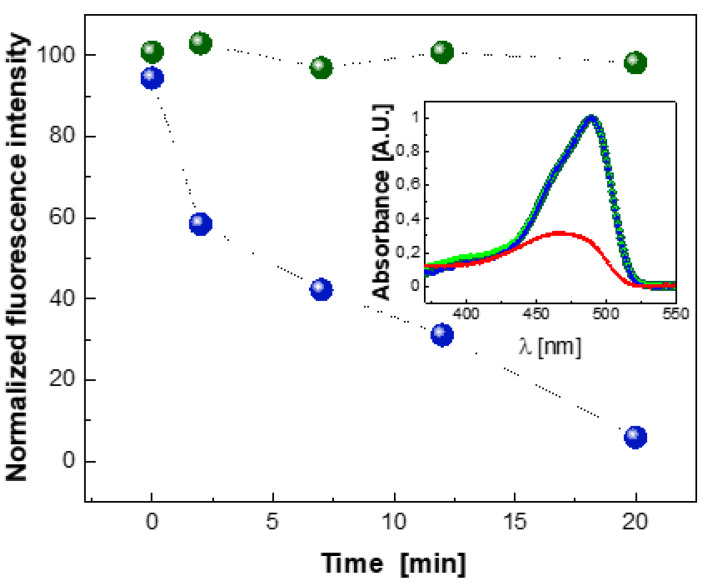
ROS-induced photobleaching of the EGFP chromophore. For the purpose of exposure of EGFP to photosensitized ROS, a 10-μM solution of EGFP in phosphate buffer (pH 8.0, 300 mM NaCl), also containing 100 μM MB and 50 mM TMP-OH (blue dots), was prepared. Such a solution and its control version (without MB and TMP-OH, green dots) were exposed to visible light during consecutive time periods of 0, 2, 7, 12, and 20 min. For the fluorescence measurement at λ_em_ = 510 nm (λ_exc_ = 489 nm), the withdrawn samples were additionally diluted to obtain the final concentration of 0.5 μM EGFP. Inset: Control absorption spectra collected for EGFP in the absence of MB and TMP-OH before (dark green trace) and after (light green trace) the exposure for 20 min to visible light; absorption spectra collected for EGFP in the presence of MB and TMP-OH before (blue trace) and after (red trace) the exposure to visible light for 20 min. The overlapping of dark green, light green, and blue lines shows that the chromophore is not bleached.

As can be seen in Figure 7, in the absence of a photosensitizer (MB), the EGFP fluorescence emission was not attenuated during the exposure to visible light for 20 min (green dots in Figure 7). In contrast, in the presence of MB, the photobleaching of the EGFP chromophore was clearly visible (blue dots in Figure 7). In fact, after 20 min of illumination the intensity of the EGFP fluorescence emission in the range 500–550 nm decreased significantly.

The relevant absorption spectra recorded before illumination and after a 20 min exposure to visible light in the presence and absence of the MB photosensitizer are shown in the inset in Figure 7.

It has to be stressed that the marked photobleaching of the EGFP chromophore reported herein was observed using MB as a photosensitizer at a relatively high concentration (100 μM) and under strong intensity of illumination with visible light.

One of the possible reasons for such a marked change in the functioning of the EGFP chromophore in the herein reported experiment using MB may actually be related to the cationic form of this photosensitizer. The cationic dye (MB) may interact with anionic EGFP chromophore by electrostatic attraction, thus resulting in an altered (impaired) state of the chromophore.

## 3. Discussion

In regard to naturally occurring fluorescent proteins and their synthetic mutants, the photoprotection and photobleaching mechanisms, as well as the potential toxicity due to a plausible photosensitization of ROS, have been the topics of intense debate.

Some evidence suggests that the function of fluorescent proteins in nature might be related to photoprotection of their hosts [38]. Concentration of fluorescent proteins in marine organisms depends on light intensity and is correlated with reduction of photodamage. Palmer et al. [9] showed that coral fluorescent proteins have significant H_2_O_2_ scavenging activity. They observed such behavior for cyan (CFP), green (GFP), red (RFP), and chromoprotein (CP) from corals.

In this context it is worth mentioning that the research concerning the protective role of GFP has been carried by F. Bou-Abdallah et al. using ESR spectroscopy. They have shown that GFP quenches O_2_^•−^ and exhibits SOD-like activity by competing with cytochrome *c* for reaction with O_2_^•−^ [39]. However, in contrast to our study, in which ROS was generated by light (either UVA or visible), F. Bou-Abdallah et al. used a standard enzymatic reaction involving xanthine oxidase, which catalyzes the oxidation of hypoxanthine to xanthine and uric acid, the process that reduces molecular oxygen (O_2_) and produces O_2_^•−^. Their investigations lead to the conclusion that GFP from jellyfish, *Aequorea victoria*, quenches superoxide radicals (O_2_^•−^) and exhibits SOD-like activity. Greenbaum et al. [40] claim that the oxidation of GFP and EGFP by O_2_^•−^ causes the photobleaching of these proteins. In line with these conclusions, Grigorenko et al. [41], using molecular modeling calculations, have shown that the light-induced reaction of GFP with oxygen leads to the formation of a radical pair, ‘chromophore^•^–O_2_^•−^’, which is then followed by the chromophore decomposition and irreversible photobleaching.

On the other hand, it was claimed, based on investigation of the GFP chromophore damage in COS7 kidney cells and in *E. coli* bacteria, following light irradiation, that GFP could generate singlet oxygen, albeit ineffectively [40]. Other reports on ROS photogeneration by GFPs were also published. In particular, time-resolved detection of the NIR luminescence was used to observe singlet oxygen (^1^Δ_g_) generation by several GFP mutants, including EGFP [42,43]. According to the authors, this effect is much stronger for the free chromophores than for the related proteins, because the protein’s β-barrel provides shielding of the chromophore from oxygen, thus reducing the possibility of photosensitization of ROS. The accessibility of molecular oxygen to the chromophore seems to play a major role in the ability of GFP-related proteins to photosensitize ^1^Δ_g_. 

Ganini et al. [44] have shown that, in contrast to the mature EGFP, the immature EGFP generates superoxide radicals (O_2_^•−^) in the presence of NADH.

Overall, the results of our research are in agreement with the reports that claim that the mature EGFP cannot generate ROS. Throughout this study, to monitor the ROS interception by EGFP, we implemented ESR in combination with spin-trapping.

In particular, we demonstrated that in the system containing solely EGFP as a potential ROS photosensitizer and DMPO or TMP-OH as a spin-traps, the ROS-related increase of the ESR signal during visible light illumination were not observed.

Our findings also support the hypothesis that GFP-like fluorescent proteins could be capable of exerting photoprotective effects in living systems. In particular, in our in vitro study, we checked the photoprotective role of EGFP in the aqueous environment using two different organic photosensitizers, RB and MB, as well as an inorganic photocatalyst, nano-TiO_2_. Such an approach enabled us to expose EGFP to a wide spectrum of ROS, ranging from singlet oxygen (^1^Δ_g_), in the case of visible light excited RB and MB, to superoxide (O_2_^•−^) and hydroxyl (OH^•^) radicals, in the case of UVA light excited nano-TiO_2_. 

In the present work, we also compared the ROS quenching capability of EGFP with the relevant abilities of three selected proteins, i.e., purine nucleoside phosphorylase (PNP), bovine serum albumin (BSA), and papain. Interestingly, the ROS scavenging role of EGFP was found similar to that of BSA and papain, whose protective activity against ROS has been suggested in the literature [34,35,36].

In the experiment, where ROS were generated by nano-TiO_2_ under exposure to UVA, the ESR signal intensity, confirming the ROS generation, decreased in the presence of EGFP as much as for BSA of the same concentration. 

While using RB as a visible light sensitive photogenerator of ROS, we found that both EGFP and papain intercept singlet oxygen (^1^Δ_g_) and superoxide radicals (O_2_^•−^) with similar efficiencies.

In addition, the spectral analysis of the ESR signals acquired during RB-mediated photosensitization of ROS under a visible light illumination suggests that EGFP scavenges O_2_^•−^ more efficiently than ^1^Δ_g_ molecules. In particular, in the presence of 10 μM EGFP, the superoxide radical-dependent component, TEMPONE, was quenched by a factor of 3.6, whereas the singlet oxygen (^1^Δ_g_)-dependent component, TEMPOL, was quenched only by a factor of 1.25 (Supplementary Figure S2).

One of the experiments performed in this work also provided evidence for the photobleaching of the EGFP chromophore under photo-oxidative stress in the presence of MB as a photosensitizer. This observation is consistent with some earlier reports [40,41,45].

In principle, such light-induced photobleaching, observed for GFP-like proteins in the presence of molecular oxygen [41], can also be caused by the photosensitized singlet oxygen. Thus, the mechanism of singlet oxygen scavenging by proteins from the GFP family might present in marine organisms, such as corals and jellyfish, and serve for photoprotection.

While elucidation of the detailed mechanism of this ROS-scavenging action of EGFP is beyond the scope of this study, the data presented herein suggest that similar protective capabilities of the naturally-occurring GFP-like proteins, such as their protective action against visible- or UV-light induced ROS, could be an important contribution to the realm of antioxidant defenses developed by, e.g., symbiotic cnidarians. In particular, it is known that these organisms experience constant pro-oxidative conditions during the daytime [46]. We also draw attention to the fact that the ability of GFP-like proteins to quench ROS should also be taken into account in other areas, where such fluorescent proteins are being implemented as supposedly inert, non-reactive light-excited fluorescent markers, e.g., in fluorescence microscopy or spectroscopic techniques, such as fluorescence correlation spectroscopy.

## 4. Materials and Methods

### 4.1. Chemicals and Protein

The following chemicals were of a reagent grade and were obtained from commercial sources: components of phosphate buffer (NaH_2_PO_4_ and Na_2_HPO_4_) and NaCl from Roth, 2,2,6,6-tetramethyl-4-piperidinol (TMP-OH), 5,5-dimethyl-1-pyrroline N-oxide (DMPO), rose bengal (RB), methylene blue (MB), superoxide dismutase from bovine erythrocytes (SOD, MW (dimer) = 32.5 kDa [47]), bovine serum albumin (BSA, MW (dimer) = 66.5 kDa [48]), and papain from papaya (MW = 23.4 kDa [49]) from Sigma-Aldrich (St. Louis, USA), nano-TiO_2_, AMT-100 (anatase) from Tayca Corp., Japan. Recombinant PNP from *E. coli* (PNP, MW (hexamer) = 6 × 25.8 = 154.8 kDa [50] was expressed in *E. coli* and purified according to the procedures described earlier [51]. To calculate the concentrations of proteins, the following extinction coefficients were used: ε_280nm_ = 57.6 mM^−1^cm^−1^ for papain [52]; ε^%^_280nm_ = 2.7 for PNP [53]. PNP concentration was calculated per monomer.

cDNA of S65T/F64L-GFP (EGFP) with 6 × His-tag was prepared using a Stratagene QuickChange mutagenesis kit. Protein was obtained by overexpression in *E. coli* strain BL21 (DE3) and purified by IMAC methods on a Ni-NTA column as described earlier [54]. The EGFP concentration was measured on a UV–VIS Cary 100Bio spectrophotometer (Varian, Agilent Technologies, Wood Dale, IL, USA) using extinction coefficient ε_280nm_ = 21.0 mM^−1^cm^−1^ for absorption of the natural aromatic amino acids residues at 280 nm and using ε_489nm_ = 38.0 mM^−1^cm^−1^ for the maximum absorption of the chromophore at 489 nm [21]. Concentrations calculated using these two extinction coefficients were the same, which means that all EGFP biomolecules have a properly maturated chromophore, and this allowed us to determine the purity of the protein as 100%.

All solutions were prepared with ultrapure water (18.2 MΩ∙cm; Millipore Sigma Simplicity^TM^ water purification system, Thermo Fisher Scientific, Basel, Switzerland).

### 4.2. Electron Spin Resonance Spectroscopy

To monitor the photogenerated ROS in the presence or absence of EGFP and other proteins used in this study, we implemented electron spin resonance (ESR) in combination with reactive spin-trapping [55,56,57]. 

The solutions prepared in 50 mM phosphate buffer (pH 8.0 with 300 mM NaCl) contained ROS photogenerators, i.e., either 100 μM MB or 50 or 100 μM RB, or 3.2 mg/mL of nano-TiO_2_ (AMT-100) ROS scavengers (50 mM TMP-OH or 10–50 mM DMPO), and EGFP. The solutions, with the standardized sample volume of 2.0 mL, contained different concentrations of EGFP, that is, of 0, 2, 5, 10, and 20 μM and of 0, 10, and 20 μM, for RB and for MB, respectively, and either of 0 or 3.6 μM for nano-TiO_2_.

The control measurements of spin concentration after 20 min of visible light illumination (150 W, halogen source) of water solutions, which contained 50 μM of rose bengal and 50 mM TMP-OH, and one of the proteins, papain (32 μM), PNP (40 μM), or EGFP (40 μM), were performed. A control measurement was performed for a solution containing no proteins. 

Prior to illumination, 2 mL volumes of the solutions/suspensions were transferred into 5 mL glass beakers. During exposure to light, the 2 mL volumes of solutions/suspensions were equilibrated with air at the atmospheric pressure and stirred vigorously (magnetic stirring) to prevent precipitation of compounds or agglomeration of nanoparticles. To avoid overheating by light, the solutions/suspensions were maintained at a stabilized temperature of 25.0 ± 0.1 °C, using a bath vessel, model Haake K10 (Thermo Fisher Scientific, Switzerland), equipped with a temperature control module.

The solutions were illuminated with the visible light from a spot halogen light source (150 W), model VOLPI 6000-1 (Intralux, Switzerland). For spin-trapping measurements of ROS generated in aqueous suspensions of nano-TiO_2_ and with using DMPO as a ROS scavenger, a UV spot light source (λ_exc_ = 365 nm, 20 mW/cm^2^), model LC-8 Lightingcure™ (Hamamatsu Photonics, Japan) was implemented.

For exposure to light, the selected consecutive time intervals of 0, 2, 7, 12, and 20 min for MB and RB and of 0, 30, 60, and 90 s for TiO_2_, were used. After each illumination step, ~15 µL sample volumes were drawn from the beaker into thin-walled borosilicate glass capillaries, model CV7087-100 (0.7 mm ID/0.87 mm OD, VitroCom Inc., Mountain Lakes, NJ, USA), which were then sealed on both ends with a tube sealant, ChaSeal (Chase Scientific Glass Inc., Rockwood, TN, USA). Next, for the subsequent ESR analysis, a thin-walled borosilicate glass capillary containing the collected sample was inserted into a standard ESR quartz tube, model 707-SQ-250M (4.0 mm OD/3.0 mm ID, Wilmad-LabGlass, Vineland, NJ, USA), and then positioned in the ESR cavity. A Bruker X-band spectrometer model ESP300E (Bruker Biospin GmbH, Karlsruhe, Germany) equipped with a standard TE102 rectangular resonator was employed for acquiring the ESR spectra. All ESR measurements of the signal growth of paramagnetic products formed from the corresponding diamagnetic precursors, that is of TEMPOL/TEMPONE (converted from TMP-OH), or of DMPO-OH/DMPO-OOH (converted from DMPO), as well as of time-dependent photo-decomposition of TEMPOL, were performed at room temperature. Typical instrumental parameters were as follows: microwave power of 10.0 mW, modulation amplitude of 0.5 G, time constant of 41.0 ms, magnetic field sweeping range of 120 G, sweeping time of 84.0 s. For each ESR spectrum, 3 or 5 traces were acquired and averaged.

### 4.3. Photobleaching of EGFP

Measurements of the characteristic fluorescence of the EGFP protein were performed to check whether the attack of ROS can photobleach this protein. To this end, 10 μM protein sample solution (in 50 mM phosphate buffer, pH 8.0, 300 mM NaCl), containing also 100 μM of MB and 50 mM of TMP-OH, was prepared. The solution prepared this way was then exposed to the visible light illumination (lamp model Haloline Eco, Osram (64695), 120 W, temperature of the halogen filament 2900 K) during the consecutive time periods of 0, 2, 7, 12, and 20 min. Next, prior the fluorescence measurements, due to the very high intensity of EGFP fluorescence, which was exceeding the measuring range of the Perkin Elmer LS 55 spectrofluorometer, a small volume (65 μL) was taken from the illuminated sample and diluted to the final EGFP concentration of 0.5 μM. Fluorescence for λ_exc_ = 489 nm and λ_em_ = 500–550 nm was measured as a function of exposure time to the visible light illumination. The results were compared with the control performed in this same way, but for the solution without MB and TMP-OH.

## Data Availability

The data presented in this study are openly available in RepOD, repod.icm.edu.pl/dataverse/repod at doi:10.18150/BXKBGY.

## Supplementary Materials

The following are available online at https://www.mdpi.com/article/10.3390/ijms22168565/s1, Supplementary Figure S1: Photogeneration of ^1^Δ_g_ and O_2_^•−^ in the absence of proteins: simulation of the ESR spectrum acquired after 20 min of illumination (VIS light, 50 μM rose bengal and 50 mM TMP-OH). Supplementary Figure S2: The spectral analysis of the photoprotective function of EGFP in an aqueous environment in vitro in the presence of ROS photogenerated by an organic photosensitizer, rose bengal, and ROS spin-trapping-TMP-OH. Supplementary Figure S3: Photogeneration of singlet oxygen (^1^Δ_g_) and superoxide radicals (O_2_^•−^) in H_2_O by 50 μM rose bengal under VIS light illumination in the absence and presence of four proteins, EGFP, PNP, papain, and BSA.

## Appendix A. Properties of the Employed ROS Photogenerators and Spin-Traps

In this work, ROS were photogenerated in aqueous solutions using organic photosensitizers, rose bengal (RB) and methylene blue (MB), or in aqueous suspensions of the inorganic photocatalyst nanocrystalline titanium dioxide (nano-TiO_2_). The potential of EGFP to photogenerate ROS was also tested. Scheme A1 summarizes the most important steps towards the formation of ROS that occur upon excitation of the RB and MB or nano-TiO_2_, with visible or UV light, respectively.

As shown in Scheme A1a, after excitation with visible light, the RB molecule passes through the intersystem-crossing process (ISC) to the long-lived excited triplet state (^3^RB*). In the subsequent reaction step, ^3^RB* transfers its energy to molecular oxygen (^3^O_2_) via the triplet–triplet energy transfer mechanism (TET), thus forming singlet oxygen (^1^Δ_g_). In addition, it has been shown that the RB molecule in its excited triplet state (^3^RB*) can also directly react with oxygen molecules (^3^O_2_) by means of the electron-transfer process (ET), thus leading to the formation of O_2_^•−^. The respective photogeneration efficiencies of formation of ^1^Δ_g_ and O_2_^•−^ have been shown to be ~75% and ~20% (Scheme A1a) [58].

The mechanism of ^1^Δ_g_ photogeneration by MB (Scheme A1b) is identical to that presented above for RB. Additionally, it has been found that the formation superoxide radicals (O_2_^•−^) may occur in the presence of significant MB concentrations ([MB] > 20 μM) [59].

In the case of the third ROS generation system used in this work (nano-TiO_2_), the absorption of the UV radiation leads to the excitation of electrons in the conduction band, e_CB_^−^, and positively charged holes in the valence band, h_VB_^+^ (Scheme A1c). The photogenerated electrons can be transferred from the conduction band of the TiO_2_ nanoparticle to the surrounding oxygen molecules, thus forming of superoxide radicals (O_2_), whereas the photogenerated holes in the valence band can react with adsorbed OH^−^ or H_2_O to form highly reactive hydroxyl radicals (OH^•^) or hydrogen peroxide (H_2_O_2_).

To monitor the photogeneration of ROS, we implemented the ESR technique in combination with spin-trapping. Spin traps can serve as efficient ROS scavengers to produce more stable spin-adducts, thus facilitating the ESR detection of short-lived forms of ROS. The molecular structures of the chosen two diamagnetic precursors, that is the nitroxide radical precursor TMP-OH and the DMPO spin-trap, are presented in Scheme A2 together with their basic reactions with ROS. As a result of these reactions, both TMP-OH and DMPO are converted to their corresponding paramagnetic forms and therefore become easily detectable by ESR.

In particular, as shown in Scheme A2a, the diamagnetic (ESR-silent) scavenger TMP–OH (2,2,6,6-tetramethyl-4-piperidinol), upon reaction with singlet oxygen (^1^Δ_g_) produces a stable paramagnetic radical product, i.e., 4-hydroxy-2,2,6,6-teramethyl piperidin-1-oxyl, TEMPOL [60].

The second ROS scavenger, the spin-trap DMPO, reacts with both OH^•^ and O_2_^•−^, thus leading to the creation of two distinguishable spin-adducts, i.e., DMPO-OH and DMPO-OOH, which clearly differ in their ESR spectra (Scheme A2b). Specifically, the corresponding ESR spectra of both paramagnetic products consist of four (DMPO-OH) and twelve (DMPO-OOH) lines. It has to be mentioned, however, that although DMPO efficiently scavenges O_2_^•−^, the resulting spin-adduct is not very stable and quickly decomposes by a direct reduction to the OH^•^ adduct, DMPO-OH (on a time scale of tens of seconds) [61]. The other possible pathway to decomposition of DMPO-OOH runs through the formation of free OH^•^ from DMPO-OOH, which can subsequently be trapped by the unreacted DMPO molecules [62].

The third ROS scavenger, TEMPOL, is a well-known, water-soluble antioxidant, reportedly acting as a superoxide dismutase (SOD) mimicking agent [63,64]. It has also been reported that TEMPOL, apart from dismutation of O_2_^•−^, can be used as a target molecule for the photogenerated OH^•^ [65]. As shown in Scheme A2c, the reported reactive pathways of TEMPOL with O_2_^•−^ and OH^•^ introduce respective structural changes at the 1- and 4-positions of the TEMPOL molecule [32,37]. Acting in concert, these structural changes induce both the decay of the paramagnetic TEMPOL, as well as the concurrent formation of another ESR-active radical, i.e., TEMPONE (4-oxo-2,2,6,6-tetramethylpiperidine-N-oxyl) [66].
