# Supplementary material for: Chromophore of an Enhanced Green Fluorescent Protein Can Play a Photoprotective Role Due to Photobleaching
Source: Int J Mol Sci. 2021 Aug 9;22(16):8565. doi: 10.3390/ijms22168565 (PMC8395242; doi:10.3390/ijms22168565)
Supplement: Supplementary file 1 [file ijms-22-08565-s001.zip › ijms-1285451-supplementary.pdf]

Supplementary Materials

1. Photo-generation of $^1\Delta_g$ and $O_2^{\bullet-}$ in the absence of proteins: simulation of the ESR spectrum acquired after 20 min of illumination (VIS light, 50 µM Rose Bengal & 50 mM TMP-OH)

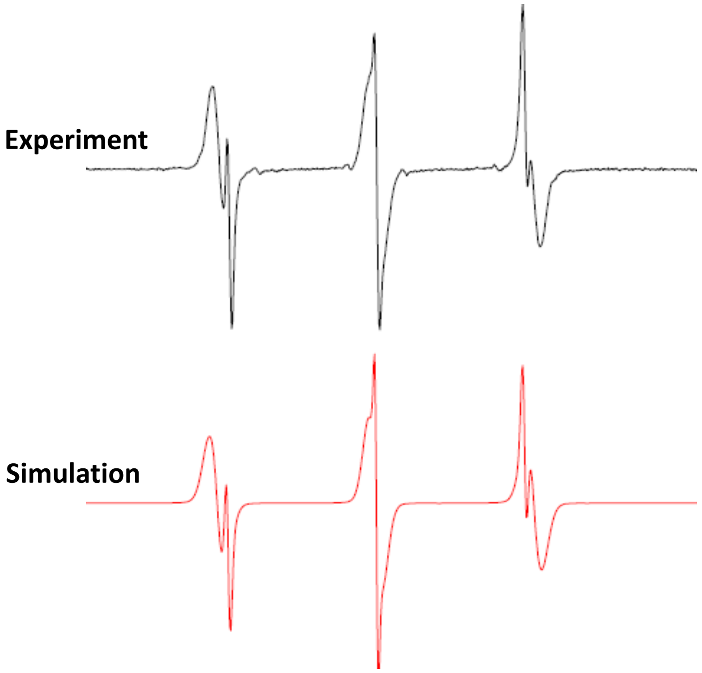

Figure S1. Simulation of the ESR signal collected for the control measurement of photogeneration of ROS ($^1\Delta_g$ and $O_2^{\bullet-}$) in water solution of Rose Bengal (50 **µM**), containing TMP-OH spin-trap (50 mM), after 20 min of illumination with visible light, in the absence of proteins and presented in the main text in Figure 6(a): the experimental trace (upper) and the simulated trace (lower). The spectral simulation points to the relative contents of 90% and 10%, for TEMPOL and TEMPONE, respectively

Spectral simulation based on summing two spectra, *i.e.,* of TEMPOL and TEMPONE, suggests that the measured experimental spectrum corresponds to the relative percentage of ~90 and ~10 percent of TEMPOL and TEMPONE, respectively.

Although this result is very close to the expected relative quantum yields of ~75 and ~20 percent for generation of singlet oxygen and superoxide radical, respectively, for Rose Bengal in H_2O, it should be accessed carefully. In particular, one should be aware that the course of reactions leading to the formation of TEMPONE is quite complicated, and that, in principle, the TEMPOL generation process, carried out by reacting TMP-OH molecules with singlet oxygen, remains the most probable process under the experimental conditions used herein.

2. **Spectral analysis of the photoprotective function of EGFP in an aquesous environment *in vitro* in the presence of ROS photo-genrated by an organic photosensitizer (Rose Bengal) – ESR spin-trapping with TMP-OH.**

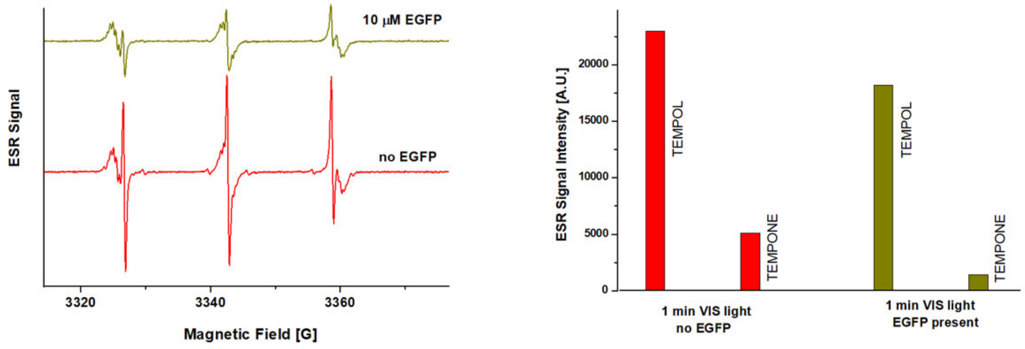

Figure S2. (left panel) Typical ESR spectra observed during RB-mediated photosensitization of ROS in the presence and absence of EGFP. (right panel) ESR signal intensities for two components of the complex ESR spectrum, *i.e.,* TEMPOL and TEMPONE, after 1 min of a visible-light illumination in the absence and presence of 10-μM EGFP. In this experiment, the photo-generated ROS in the presence of 10-μM RB were scavenged by a spin-trap, TMP-OH (50 mM) in 50 mM phosphate buffer pH 8.0

The spectral analysis of the ESR signals acquired during RB-mediated photosensitization of ROS under a visible-light illumination suggests that EGFP scavenges $O_2^{\bullet -}$ radicals more efficiently than $^1\Delta_g$ molecules. In particular, in the presence of 10 μM EGFP, the superoxide radical ($O_2^{\bullet -}$) - dependent component, TEMPONE, was quenched by a factor of 3.6, whereas the singlet oxygen ($^1\Delta_g$) - dependent component, TEMPOL, was quenched only by a factor of 1.25.

3. **Photo-generation of singlet oxygen ($^1\Delta_g$) and superoxide radicals ($O_2^{\bullet-}$) in H_2O by 50 mM Rose Bengal under VIS light illumination in the absence and presence of four proteins, EGFP, PNP, papain and BSA.**

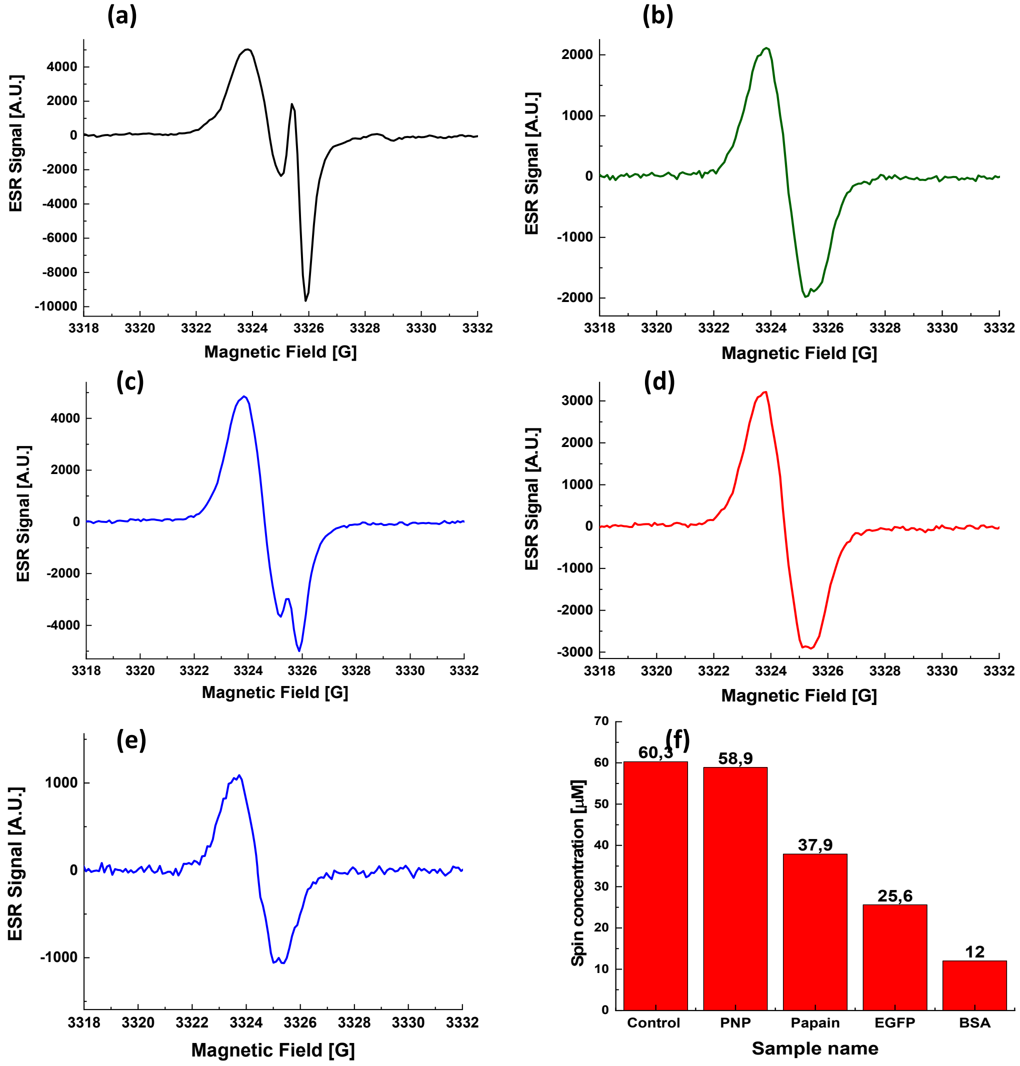

Figure S3. Comparison of the ROS quenching capacity of four proteins, EGFP, PNP, papain and BSA, during the photosensitized generation of singlet oxygen ($^1\Delta_g$) and superoxide radicals ($O_2^{\bullet-}$) by 50-µM Rose Bengal under exposure to visible light illumination. Under the action of photosensitized ROS, the spin-trap TMP-OH (50-mM) is converted to the ESR-active nitroxide radicals, TEMPOL and TEMPONE. (a) The low-field hyperfine ESR feature acquired for the control measurement in the absence of proteins. (b) The low-field hyperfine ESR feature acquired in the presence of 40-µM EGFP. (c) The low-field hyperfine ESR feature acquired in the presence of 40-µM PNP. (d) The low-field hyperfine ESR feature acquired in the presence of 32-µM papain. (e) The low-field hyperfine ESR feature acquired in the presence of 40-µM BSA. (f) Juxtaposition of detected spin concentrations calculated from signal intensities of ESR signals recorded for four tested proteins after 20 min of exposure to visible light. All ESR spectra were recorded using the same experimental settings (0.63 mW microwave power, 0.5 G mod., 4 scans per trace).

To demonstrate the ROS quenching ability of EGFP in comparison to other proteins, a measurement of ROS scavenging by four selected proteins, EGFP, PNP, papain and BSA, was performed. Specifically, in this experiment, ROS were photo-generated by an organic photosensitizer, Rose Bengal (RB), under exposure to visible light. In this context, it is widely accepted that, in aqueous milieus, RB, which is a good photosensitizer of singlet oxygen ($^1\Delta_g$), generates also superoxide radicals ($O_2^{\bullet-}$), at proportions of 75% and 20%, respectively (see Scheme A1(a)).

In order to detect the photo-sensitized ROS in the presence and absence of the selected proteins, we used a combination of ESR with spin-trapping. An ESR-silent scavenger, TMP–OH (2,2,6,6-tetramethyl-4-piperidinol), was used as a spin-trap of ROS in this experiment. Upon reaction with $^1\Delta_g$, TMP-OH is converted to a paramagnetic radical product, *i.e.,* 4-hydroxy-2,2,6,6-teramethyl piperidin-1-oxyl, TEMPOL [1].

Although TEMPOL is relatively stable, it can react with certain short-lived radicals, like, *e.g.,* hydroxyl radicals ($OH^{\bullet}$) [2].

As it is shown in Scheme A2(c), the reported reactive pathways of TEMPOL with OH• (a byproduct of photosensitization of $O_2^{\bullet-}$) introduce structural changes at the 1- and 4-positions of the TEMPOL molecule [3]. These structural changes induce both the decay of the paramagnetic TEMPOL, as well as the concurrent formation of another ESR-active radical*, i.e.,* TEMPONE (4-oxo-2,2,6,6-tetramethylpiperidine-N-oxyl) [4].

Therefore, with the simultaneous presence of $^1\Delta_g$ singlet oxygen and $O_2^{\bullet-}$, a complex signal consisting of TEMPOL and TEMPONE components is observed. This can clearly be seen in Figure S3(a), which shows the low-field hyperfine feature of the ESR signal observed during photo-generation of ROS by RB. The narrow and slightly shifted toward higher magnetic fields TEMPONE component is easily distinguishable from the wider signal of TEMPOL. Thus, the resulting signal shown in Figure S3(a), collected after 20 min of illumination with visible light, can be considered a reference for subsequent experiments using selected proteins as ROS scavengers.

In fact, as shown in Figures 3S (b)-(e), both the intensity of the observed ESR signals and their spectral shapes change in the presence of four proteins. Specifically, in the presence of proteins, the overall ESR signal intensities decay, which may indicate trapping of ROS by proteins. In addition, depending on the protein, a differentiated disappearance of the TEMPONE component can also be seen.

Figure 3S(f) shows the resultant tendency of the ESR signal disappearance for four selected proteins herein studied. The shown trend can be understood as a comparison of the ROS-quenching abilities of these proteins. Thus, the dependence shown in **Figures 3S (b)-(e)**, suggests that the ROS quenching ability runs from the highest to the lowest in the following way: BSA > EGFP > papain >PNP.

[1] Lion, Y., Delmelle, M., Van de Vorst, A. 'New method of detecting singlet oxygen production', *Nature* **1976**, *263,* 442–443. doi: 10.1038/263442a0].

[2] Saito, K., Takeshita, K., Ueda, J., Ozawa, T. Two reaction sites of a spin label, TEMPOL (4-hydroxy-2,2,6,6-tetramethylpiperidine-N-oxyl), with hydroxyl radical*. J Pharm Sci.* **2003**, *92*, 275-280. doi: 10.1002/jps.10304

[3] Marshall, D.L., Christian, M.L., Gryn'ova, G., Coote, M.L., Barker, P.J., Blanksby, S.J. 'Oxidation of 4-substituted TEMPO derivatives reveals modifications at the 1- and 4-positions', *Org Biomol Chem.* **2011**, *9*, 4936-4947. doi: 10.1039/c1ob05037k

[4] Kudo, W., Yamato, M., Yamada, K., Kinoshita, Y., Shiba, T., Watanabe, T., Utsumi, H. Formation of TEMPOL-hydroxylamine during reaction between TEMPOL and hydroxyl radical: HPLC/ECD study. *Free Radic Res.* **2008**, *42*, 505-512. doi: 10.1080/10715760802112809
